# Prognostic significance of programmed cell death ligand 1 blood markers in non-small cell lung cancer treated with immune checkpoint inhibitors: a systematic review and meta-analysis

**DOI:** 10.3389/fimmu.2024.1400262

**Published:** 2024-06-10

**Authors:** Ningning Zhang, Jianlan Chang, Ping Liu, Xiangyang Tian, Junyan Yu

**Affiliations:** Department of Oncology, Heping Hospital Affiliated to Changzhi Medical College, Changzhi, Shanxi, China

**Keywords:** non-small cell lung cancer, immune checkpoint inhibitors, programmed cell death ligand 1, blood biomarker, meta-analysis

## Abstract

**Background:**

Immune checkpoint inhibitors (ICIs) are effective for non-small cell lung cancer (NSCLC) treatment, but the response rate remains low. Programmed cell death ligand 1 (PD-L1) in peripheral blood, including soluble form (sPD-L1), expression on circulating tumor cells (CTCs PD-L1) and exosomes (exoPD-L1), are minimally invasive and promising markers for patient selection and management, but their prognostic significance remains inconclusive. Here, we performed a meta-analysis for the prognostic value of PD-L1 blood markers in NSCLC patients treated with ICIs.

**Methods:**

Eligible studies were obtained by searching PubMed, EMBAS, Web of Science, and Cochrane Library prior to November 30, 2023. The associations between pre-treatment, post-treatment and dynamic changes of blood PD-L1 levels and progression-free survival (PFS)/over survival (OS) were analyzed by estimating hazard ratio (HR) and 95% confidence interval (CI).

**Results:**

A total of 26 studies comprising 1606 patients were included. High pre- or post-treatment sPD-L1 levels were significantly associated with worse PFS (pre-treatment: HR=1.49, 95%CI 1.13–1.95; post-treatment: HR=2.09, 95%CI 1.40–3.12) and OS (pre-treatment: HR=1.83, 95%CI 1.25–2.67; post-treatment: HR=2.60, 95%CI 1.09–6.20, P=0.032). High pre-treatment exoPD-L1 levels predicted a worse PFS (HR=4.24, 95%CI 2.82–6.38, P<0.001). Pre-treatment PD-L1^+^ CTCs tended to be correlated with prolonged PFS (HR=0.63, 95%CI 0.39–1.02) and OS (HR=0.58, 95%CI 0.36–0.93). Patients with up-regulated exoPD-L1 levels, but not sPD-L1, after ICIs treatment had significantly favorable PFS (HR=0.36, 95%CI 0.23–0.55) and OS (HR=0.24, 95%CI 0.08–0.68).

**Conclusion:**

PD-L1 blood markers, including sPD-L1, CTCs PD-L1 and exoPD-L1, can effectively predict prognosis, and may be potentially utilized for patient selection and treatment management for NSCLC patients receiving ICIs.

## Introduction

Lung cancer remains one of the most common malignant tumors and one of the leading causes of cancer-related death worldwide (1). Despite greatly improved prognosis attributed to molecular targeted therapy in recent decades, most of advanced non-small cell lung cancer (NSCLC) patients inevitably develop drug resistance (2, 3). As an innovative therapy, immunotherapy has become an emerging and promising treatment strategy for various cancers, especially lung cancer (4, 5). The most commonly utilized immunotherapy is immune checkpoint inhibitors (ICIs) that include blockades for programmed cell death 1 (PD-1), programmed cell death ligand 1 (PD-L1), and cytotoxic T-lymphocyte associated protein 4 (CTLA-4). However, the response rate to ICIs is still low in NSCLC, and exploring biomarkers to select patients with best clinical response and benefit and to predict prognosis is vital (6, 7).

Numerous studies have demonstrated better clinical response and superior survivals in ICIs-treated patients with overexpressed PD-L1 on tumor tissues (8). Until now, the membrane-bound form PD-L1 expressed on tumor cells is the only officially approved and well-established biomarker for patient selection (9). However, the utilization of tumor PD-L1 marker is still in controversy, which is challenged by the highly heterogeneous spatial expression pattern in tumor tissues, highly dynamic changes at different disease stages, and inconvenience of longitudinal tissue sampling owing to the invasive biopsy method (10). Therefore, novel biomarkers that are less heterogeneously expressed, can be detected via less invasive methods and has sufficient prognostic significance are in urgent need for ICIs treatment.

Besides of expression on tumor tissues, PD-L1 can be expressed and detected in peripheral blood, such as the soluble form PD-L1 (sPD-L1) in serum/plasma, PD-L1 expressed in circulating tumor cells (CTC PD-L1) and exosomes (exoPD-L1) (11–13). These PD-L1 blood markers can be less-invasively detected and then monitored for dynamic changes during treatments. They are usually overexpressed in cancer patients in association with clinicopathological features, and may predict response and prognosis to various anti-cancer treatments, such as chemotherapy, radiotherapy, targeted therapy and immunotherapy, in many types of cancers (14–16). In the scenario of NSCLC patients treated with ICIs, the predictive and prognostic values of PD-L1 blood markers have also been explored (16–18). Yet, the conclusions remain controversial due to varieties in sample size, detection methods, biomarker cut-off values, and sampling time points (19, 20). With the accumulated evidence in recent years, we performed a systematic review and meta-analysis to explore the prognostic significance of PD-L1 blood markers, including sPD-L1, CTC PD-L1 and exoPD-L1, in NSCLC patients treated with ICIs.

## Materials and methods

### Literature search

This systematic review and meta-analysis was conducted in compliance with the Preferred Reporting Items for Systematic review and Meta-analysis (PRISMA) guideline (21). Candidate studies investigating the prognostic value of blood PD-L1 markers in NSCLC patients undergoing ICIs therapy were comprehensively searched in PubMed, EMBAS, Web of Science, and Cochrane Library from inception to November 30, 2023. The following search terms were applied: (exosome OR exosomal OR extracellular vesicles OR soluble OR circulating tumor cells OR neoplastic circulating cells OR CTCs) AND (programmed cell death ligand 1 OR PD-L1 OR CD274 OR sPD-L1 OR exoPD-L1) AND (NSCLC OR non-small cell lung cancer OR lung adenocarcinoma OR lung cancer OR lung carcinoma). Language was not restricted. The reference lists of included studies and relevant reviews were manually reviewed for additional eligible articles.

### Inclusion and exclusion criteria

The eligibility of retrieved studies was judged according to the following criteria (1): NSCLC patients were treated with ICIs alone or in combination with other therapies; (2) at least one of blood PD-L1, including sPD-L1, CTC PD-L1 and exoPD-L1, were detected; (3) pre-treatment level, post-treatment level or dynamic change of blood PD-L1 was determined as prognostic marker; (4) the association of PD-L1 marker with progression-free survival (PFS) and/or overall survival (OS) was investigated. Case reports, reviews, meta-analyses, conference abstracts, and functional studies were excluded. If recruiting various cancers including NSCLC, the study was included only when survival outcomes of NSCLC subgroup were reported. Otherwise, it was discarded. Since we aimed for the prognostic value of functional PD-L1, studies detecting mRNA expression on exosomes were excluded.

### Quality assessment of included studies

Newcastle-Ottawa Scale (NOS) for cohort study was used to assess the quality of included studies (22). The risk of bias with regard to selection, comparability, and outcome was judged. Selection and outcome domains contained 4 and 3 items, respectively, and 0 or 1 score was awarded to each item. In comparability domain, 0, 1 or 2 scores were awarded. Thus, the total score ranged from 0 to 9. A study with ≥7, 4–6, and ≤5 scores were deemed to have a high, moderate, and low quality, respectively. Quality assessment was performed by two independent authors (JC, PL), and conflicts were resolved by a third author (XT).

### Data extraction

Two independent authors (JC, PL) extracted the following information of each study: first author, publication year, study design, region, cancer stage, sample size, age, percentage of male, percentage of smoker, type and treatment line of ICIs, CTC enrichment method, PD-L1 detection method, enzyme-linked immunosorbent assay (ELISA) kit, cut-off value, cut-off determination method, time point of marker detection, hazard ratio (HR) with corresponding 95% confidence interval (CI) of survival outcomes (PFS, OS), and follow-up duration. We extracted HR and 95%CI from multivariate analysis if available. Otherwise, HR results from univariate analysis were extracted. If HR was not directly reported, we estimated it after extracting the survival data from Kaplan-Meier curve if available using Engauge Digitizer v12.1. Disagreement was resolved by a third author (XT).

### Statistical analysis

The statistical analysis was conducted by using STATA 16.0 (Stata Corporation, TX, USA). The model for quantitative synthesis was determined by between-study heterogeneity. A fixed-effect model was applied when there was low heterogeneity (I^2^<50% and Q test P>0.10). Otherwise, a random-effect model was used. The association strength between PD-L1 blood markers was calculated by pooled HR with 95%CI. The prognostic values of pre-treatment, post-treatment, and dynamic change of PD-L1 blood markers in predicting PFS and OS was assessed separately. Subgroup analysis and meta-regression were performed to explore the potential source of heterogeneity. Subgroup analyses were stratified by study design, region, PD-L1 detection method, ELISA kit, CTC enrichment method, cut-off value, cut-off determination method, HR analysis, HR extraction, and sample size. Meta-regression analysis was done to explore the modulation effect of baseline variables, including median age, percentage of males, percentage of smokers, sample size, percentage of first-line immunotherapy, and cut-off value, on prognostic value of PD-L1 blood markers. Funnel plot was viewed for symmetry and Egger’s test was performed to assess potential publication bias. In case of potential publication bias, we implemented a trim-and-fill analysis to explore the impact of publication bias on pooled results. P<0.05 indicated statistical significance.

## Results

### Baseline characteristics of studies included in meta-analysis

**Figure 1:**
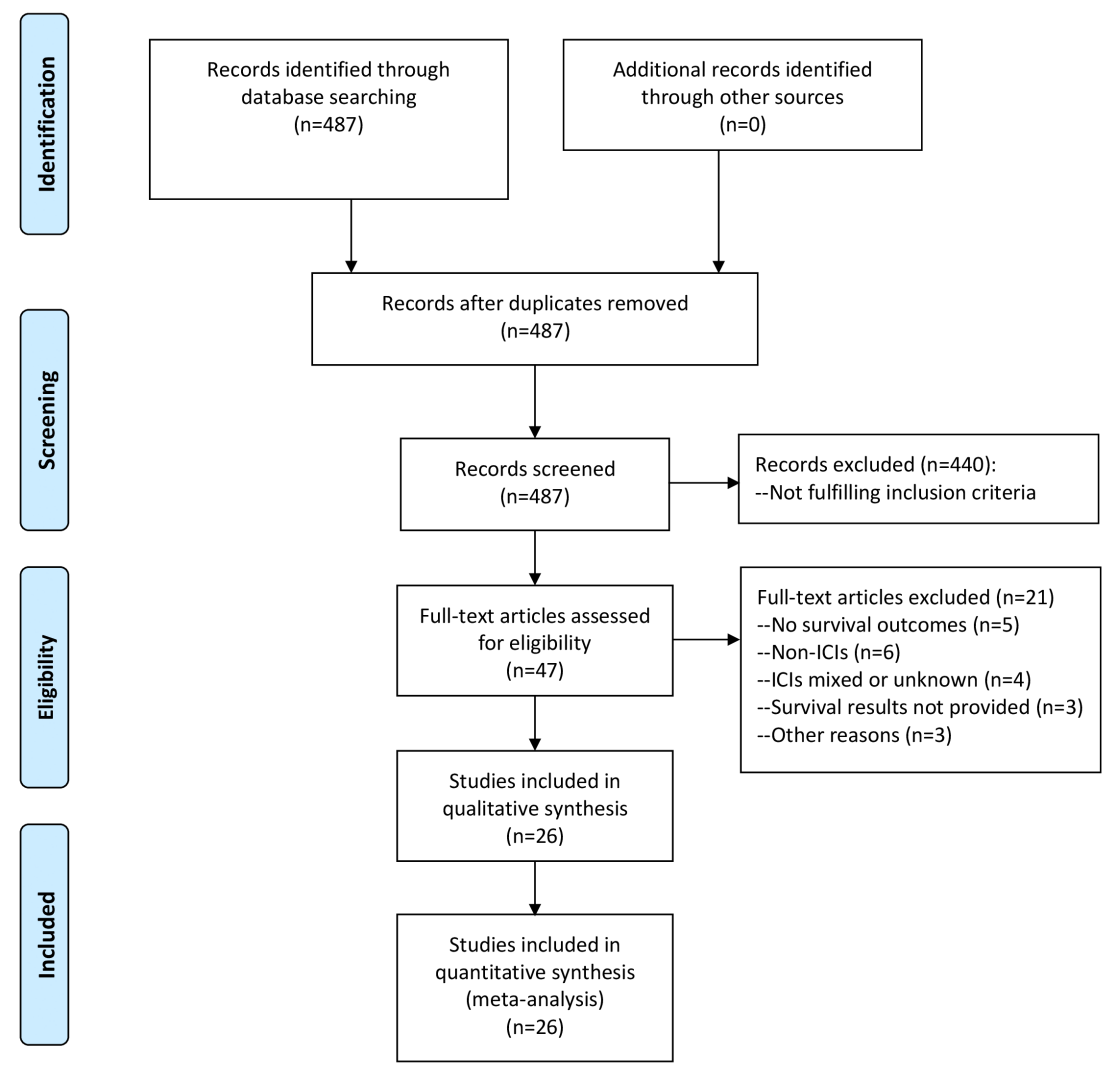
Flow chart of literature search.

As illustrated in **Figure 1**, we obtained a total of 487 articles by comprehensive literature search and discarded 440 after reviewing titles and abstracts as they did not fulfill the inclusion criteria. We further reviewed the full texts of the remaining 47 studies for eligibility and excluded 21 with the following reasons: survival outcomes not investigated (n=5), non-ICI treatments (n=6), various treatments including ICIs (n=2), ICIs treatment unknown (n=2), no NSCLC patients (n=1), survival results not provided (n=3), survival analysis at specimen level (n=1), PD-L1 mRNA expression in exosomes (n=1). These articles with detailed exclusion reasons were listed in **Supplementary Table 1**. Finally, 26 studies comprising 1606 NSCLC patients receiving ICIs treatment were included in the meta-analysis (16, 17, 19, 20, 23–44). The prognostic significance of sPD-L1, CTC PD-L1 and exoPD-L1 were investigated in 16, 9 and 3 studies, respectively. The correlations of survival outcomes with pre-treatment blood PD-L1 markers, post-treatment markers and dynamic changes from baseline were reported by 21, 5 and 7 studies, respectively. There were 4 retrospective studies and 22 prospective studies. These studies were conducted in East Asian countries (n=12), European countries (n=12) or America (n=2). The sample size ranged greatly from 14 to 233. PFS outcome was analyzed in 26 studies while OS outcome was analyzed in 19 studies. According to NOS, all included studies had high methodological quality. The baseline characteristics of all studies included in meta-analysis were summarized in **Table 1**.

**Table 1:** Baseline characteristics of studies included in meta-analysis.

Study	Design	Region	Stages	No.	Median age (range), year	%Male	%Smoker	ICI type	Treatment line	Marker	Time point	Outcome	NOS score
Dhar M, 2018 (24)	P	USA	Advanced	17	65 (59–91)	41	NA	Nivolumab, pembrolizumab, avelumab, ipilimumab	NA	CTC PD-L1	Pre-treatment	PFS	7
Guibert N, 2018 (20)	P	France	Advanced	89	60 (30–81)	66	NA	Nivolumab	2	CTC PD-L1	Pre-treatment	PFS, OS	7
Okuma Y, 2018 (27)	P	Japan	IV or recurrence	39	69 (50–88)	74	72	Nivolumab	≥ 2	sPD-L1	Pre-treatment	OS	8
Costantini A, 2018 (19)	P	France	I-II (7%), III-IV (93%)	43	68 (IQR 62–71.5)	67	88	Nivolumab	≥ 2	sPD-L1	Post-treatment, dynamic change	PFS, OS	8
Tiako Meyo M, 2020 (29)	P	France	Advanced	51	66 (IQR 60–69)	57	96	Nivolumab	≥ 2	sPD-L1	Pre-treatment, dynamic change	PFS, OS	8
Castello A, 2020 (23)	P	Italy	NA	20	76.5 (51–86)	65	NA	Nivolumab, pembrolizumab	1 (30%), ≥ 2 (70%)	sPD-L1	Pre- and post-treatment, dynamic change	PFS, OS	7
Zhang C, 2020 (16)	P	China	IV	24	NA	NA	NA	NA	NA	exoPD-L1	Pre-treatment	PFS	7
Papadaki M, 2020 (28)	P	Greece	Advanced	15	70 (61–82)	87	NA	NA	≥ 2	CTC PD-L1	Pre-treatment	PFS, OS	7
Mazzaschi G, 2020 (25)	P	Italy	IIIB (3%), IV (97%)	109	72 (41–85)	67	77	Nivolumab, pembrolizumab, atezolizumab	1 (14%), ≥ 2 (86%)	sPD-L1	Pre-treatment	PFS, OS	8
Murakami S, 2020 (26)	R	Japan	Advanced or recurrent	233	63 (30–84)	65	77	Nivolumab, pembrolizumab	1 (17%), ≥ 2 (83%)	sPD-L1	Pre-treatment	PFS, OS	9
Zizzari I, 2020 (38)	P	Italy	IV	22	NA	73	86	Nivolumab	≥ 2	sPD-L1	Post-treatment	OS	8
Dall’Olio F, 2021 (21)	P	Italy	Advanced	24	68 (53–83)	62	NA	NA	≥ 2	CTC PD-L1	Pre-treatment	PFS, OS	8
Yang Q, 2021 (35)	P	China	Advanced	21	NA	NA	NA	NA	NA	sPD-L1, exoPD-L1	Dynamic change	PFS, OS	7
Ikeda M, 2021 (30)	P	Japan	III (25%), IV (75%)	16	NA	NA	NA	Nivolumab	NA	CTC PD-L1	Post-treatment	PFS, OS	7
Oh S, 2021 (31)	R	Korea	IV	17	NA	NA	NA	Various	NA	sPD-L1	Dynamic change	PFS, OS	7
Zamora Atenza C, 2022 (36)	P	Spain	Advanced	119	65 (36–84)	78	91	NA (9% plus chemo)	1 (31%), ≥ 2 (69%)	sPD-L1	Pre-treatment	PFS, OS	8
Schehr J, 2022 (32)	P	USA	IV	14	NA	NA	NA	Nivolumab, pembrolizumab, atezolizumab	NA	CTC PD-L1	Pre-treatment	PFS	7
Wang Y, 2022 (34)	P	China	II (3%), III (23%), IV (74%)	149	NA	80	67	NA	1 (43%), ≥ 2 (57%)	exoPD-L1	Pre-treatment, dynamic change	PFS	8
Zhang Y, 2022 (37)	P	China	IIIa/IIIb (13%), IV (87%)	30	56 (48–69)	73	54	Sintilimab + docetaxel	2	CTC PD-L1	Pre-treatment	PFS, OS	8
Spliliotaki 2022	P	Cyprus	Advanced	24	66 (40–82)	81	NA	Pembrolizumab	2	CTC PD-L1	Dynamic change	PFS	7
Zhou Q, 2023 (44)	P	China	III (18%), IV (86%)	49	62 (37–77)	73	67	Various (4% plus chemo)	1 (12%), ≥ 2 (88%)	CTC PD-L1	Pre-treatment	PFS	8
Genova C, 2023 (PC) (40)	P	Italy	IV	56	70.1 (50.5–88.8)	77	93	Pembrolizumab	1	sPD-L1	Pre-treatment	PFS, OS	8
Genova C, 2023 (NC) (40)	P	Italy	IIIB (5%), IV (95%)	126	70.1 (44.2–87.6)	72	90	Nivolumab	≥ 2	sPD-L1	Pre-treatment	PFS, OS	8
Yi L, 2023 (42)	R	China	IIB-IIIC (23%), IV (77%)	39	NA	74	74	Nivolumab, pembrolizumab, sintilimab	1 (41%), 2 (59%)	sPD-L1	Pre-treatment	PFS	8
Himuro H, 2023 (41)	P	Japan	NA	122	71	75	78	Nivolumab, pembrolizumab, atezolizumab	1 (53%), ≥ 2 (47%)	sPD-L1	Pre- and post-treatment	PFS, OS	8
Zhang S, 2023 (43)	P	China	IV	18	61 (41–79)	33	16	Toripalimab + anlotinib	≥ 2	sPD-L1	Pre-treatment	PFS	8
Chmielewska I, 2023 (39)	R	Poland	IIIB (8%), IV (92%)	120	68	58	NA	Nivolumab, pembrolizumab, atezolizumab (18% plus chemo)	1 (61%), 2 (39%)	sPD-L1	Pre-treatment	PFS, OS	7

Chemo, chemotherapy; CTC, circulating tumor cell; exoPD-L1, exosomal PD-L1; ICI, immune checkpoint inhibitor; IQR, interquartile range; NA, not available; NC, nivolumab cohort; OS, overall survival; P, prospective; PC, pembrolizumab cohort; PD-L1, programmed cell death 1 ligand 1; PFS, progression-free survival; R, retrospective; sPD-L1, soluble PD-L1.

For pre- or post-treatment sPD-L1 and exoPD-L1, cut-off value of PD-L1 concentrations was determined to categorize high and low groups. For dynamic change, cut-off value of change fold was set to segregate patients with up-regulation and without up-regulation after immunotherapy. For pre- or post-treatment CTC PD-L1, cut-off value was used to define PD-L1^+^ CTCs. Seven studies determined the cut-off value by using the median value of PD-L1 concentrations. Nine studies established the cut-off value as the optimal point showing the best performance by various analyses, such as receiver operating characteristic (ROC) curve, Classification and regression tree (CART) analysis, and or log-rank test. Details regarding CTC enrichment, PD-L1 detection, cut-off determination, HR analysis and extraction, and follow-up duration were shown in **Supplementary Table 2**.

### Prognostic significance of sPD-L1

**Figure 2:**
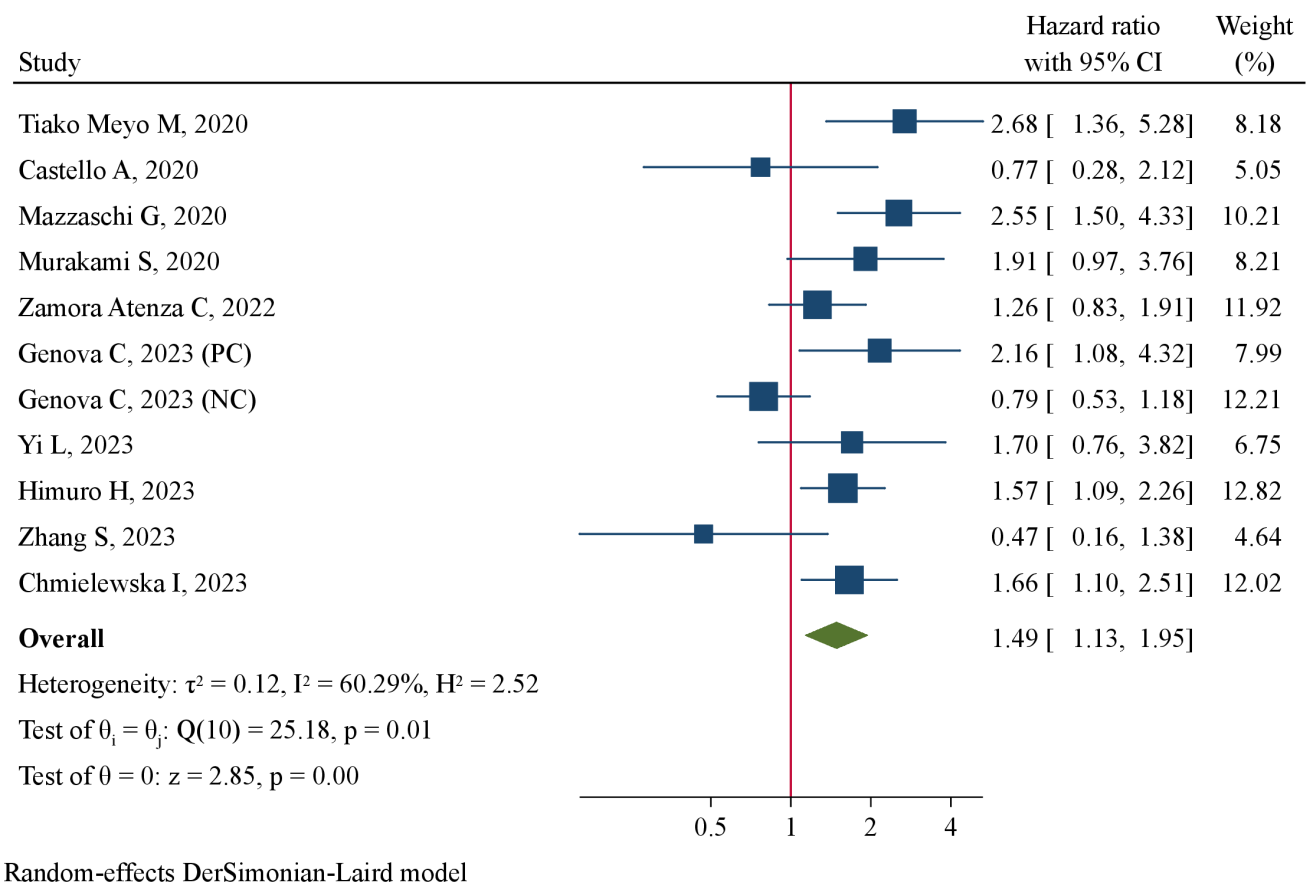
Forest plot of pre-treatment sPD-L1 levels in association with progression-free survival.

**Figure 3:**
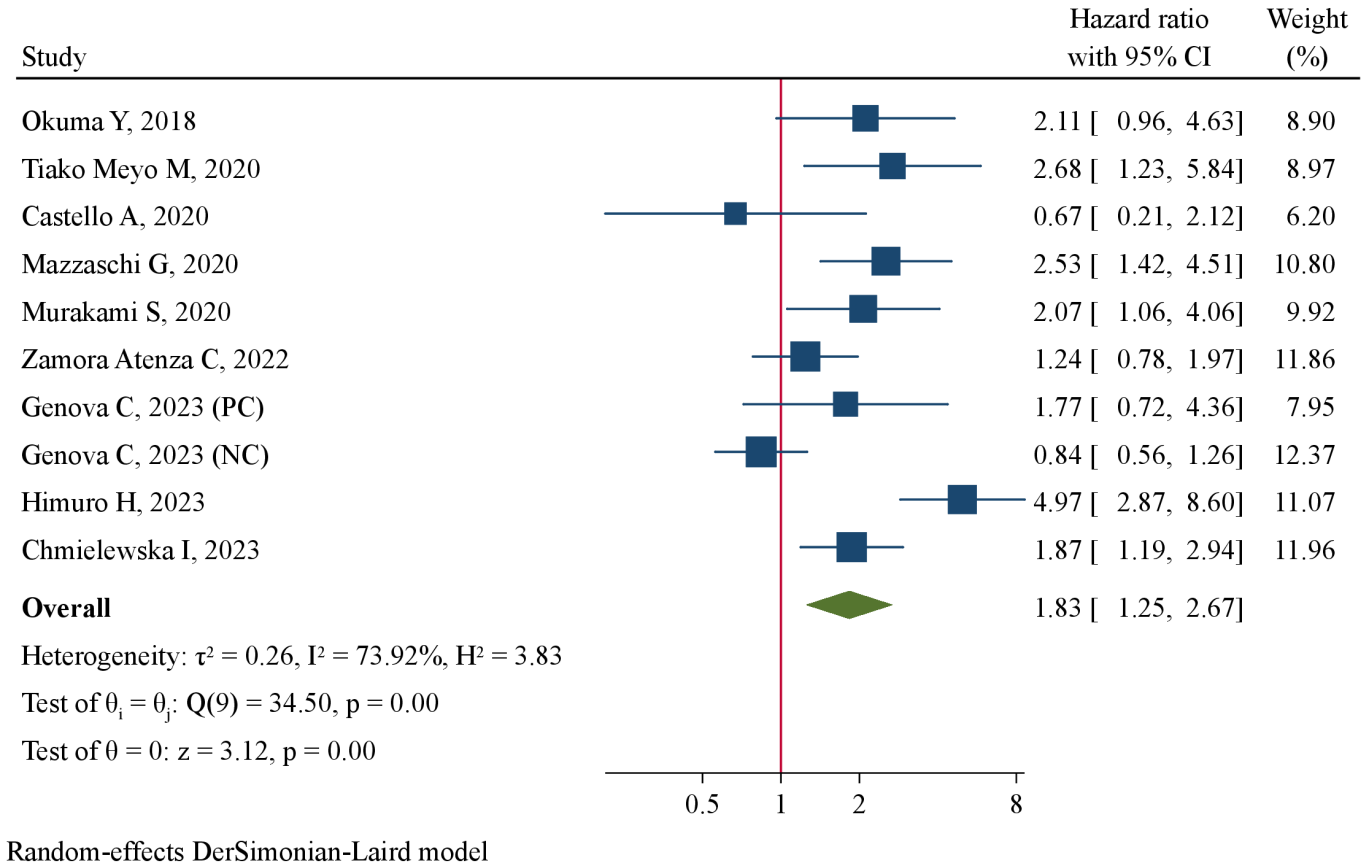
Forest plot of pre-treatment sPD-L1 levels in association with overall survival.

The association between pre-treatment sPD-L1 and PFS was investigated in 11 studies involving 1011 NSCLC patients treated with ICIs. There was substantial level of between-study heterogeneity (I^2 =^ 60.3%, P=0.005), and a random-effect model was used. Pooled analysis demonstrated patients with high pre-treatment sPD-L1 concentrations had significantly worse PFS than those with low concentrations (HR=1.49, 95%CI 1.13–1.95, P=0.004, **Figure 2**). The correlation of pre-treatment sPD-L1 with OS was analyzed in 10 studies comprising 995 patients. Using a random-effect model, meta-analysis revealed significantly shorter OS in patients with high sPD-L1 levels compared with those with low sPD-L1 levels (HR=1.83, 95%CI 1.25–2.67, P=0.002, **Figure 3**). Further analysis stratified by baseline characteristics were performed (**Table 2**). There were no significant between-subgroup differences (all P values >0.05), indicating that baseline features were not the main sources of heterogeneity.

**Table 2:** Subgroup analysis of pre-treatment sPD-L1 in association with survival outcomes.

Subgroup	PFS					OS				
	N	I^2^, %	HR (95%CI)	P1	P2	N	I^2^, %	HR (95%CI)	P1	P2
Study design					0.396					0.814
Prospective	8 (619)	70.5	1.39 (0.96–2.01)	0.079		8 (642)	79.4	1.79 (1.09–2.94)	0.021	
Retrospective	3 (392)	0	1.72 (1.25–2.38)	0.001		2 (353)	0	1.93 (1.33–2.81)	<0.001	
Region					0.822					0.073
Ease Asia	4 (410)	41.5	1.51 (1.13–2.01)	0.005		3 (394)	61.1	2.91 (1.57–5.37)	<0.001	
Europe or America	7 (601)	70.0	1.53 (1.05–2.22)	0.026		7 (601)	63.5	1.50 (1.03–2.19)	0.034	
Detection method					–					–
ELISA	10 (993)	56.8	1.57 (1.21–2.03)	<0.001		10 (993)	73.9	1.83 (1.25–2.67)	0.002	
Others	1 (18)	–	–	–		0	–	–	–	
ELISA kit					0.587					0.274
R&D systems	4 (482)	38.5	1.74 (1.34–2.26)	<0.001		4 (484)	72.8	2.32 (1.20–4.50)	0.013	
Others	6 (511)	64.1	1.50 (1.04–2.15)	0.029		6 (511)	58.0	1.52 (1.05–2.19)	0.025	
Cut-off value					0.506					0.207
<100 pg/ml	7 (794)	52.3	1.36 (1.03–1.80)	0.029		7 (796)	80.3	1.63 (0.99–2.67)	0.055	
≥100 pg/ml	4 (217)	65.4	1.72 (0.92–3.23)	0.092		3 (199)	0	2.45 (1.64–3.66)	<0.001	
Cut-off determination					0.712					0.155
Median value	5 (361)	62.6	1.31 (0.84–2.03)	0.236		4 (322)	64.2	1.22 (0.72–2.07)	0.468	
Optimal cut-off point	3 (348)	52.8	1.66 (1.16–2.38)	0.006		4 (389)	79.4	2.38 (1.26–4.51)	0.008	
Others	3 (302)	72.8	1.47 (0.62–3.53)	0.384		2 (284)	0	2.31 (1.39–3.85)	0.001	
HR analysis					0.800					0.282
Univariate	8 (596)	48.2	1.56 (1.18–2.07)	0.002		7 (580)	68.8	2.08 (1.36–3.18)	<0.001	
Multivariate	3 (415)	77.0	1.42 (0.70–2.86)	0.333		3 (415)	67.3	1.36 (0.71–2.60)	0.349	
HR extraction					0.265					0.664
Reported	7 (814)	68.4	1.65 (1.17–2.34)	0.005		7 (814)	61.8	1.65 (1.17–2.34)	0.005	
Estimated	4 (197)	48.9	1.36 (1.00–1.83)	0.049		3 (181)	81.0	2.11 (0.73–6.10)	0.167	
Sample size					0.909					0.899
<100	5 (184)	59.9	1.43 (0.79–2.56)	0.237		4 (166)	24.6	1.85 (1.20–2.86)	0.006	
≥100	6 (827)	66.3	1.48 (1.08–2.03)	0.015		6 (829)	83.6	1.89 (1.13–3.14)	0.015	

CI, confidence interval; HR, hazard ratio; N, number of studies and patients; P1, p value of pooled analysis; P2, p value for between-subgroup comparison.

Post-treatment sPD-L1 levels were determined for association with PFS and OS in 3 (153 patients) and 4 (177 patients) studies, respectively. High post-treatment sPD-L1 was significantly correlated with worse PFS (HR=2.09, 95%CI 1.40–3.12, P<0.001, **Supplementary Figure 1**) and OS (HR=2.60, 95%CI 1.09–6.20, P=0.032, **Supplementary Figure 2**) compared with low post-treatment sPD-L1.

The prognostic value of dynamic changes of sPD-L1 concentrations was evaluated in 5 studies including 139 NSCLC patients. Up-regulation of sPD-L1 levels from baseline to several cycles of immunotherapy was not significantly associated with survival outcomes (PFS: HR=0.76, 95%CI 0.47–1.23, P=0.259; OS: HR=0.68, 95%CI 0.39–1.19, P=0.117; **Supplementary Figure 3**).

### Prognostic significance of CTC PD-L1

**Figure 4:**
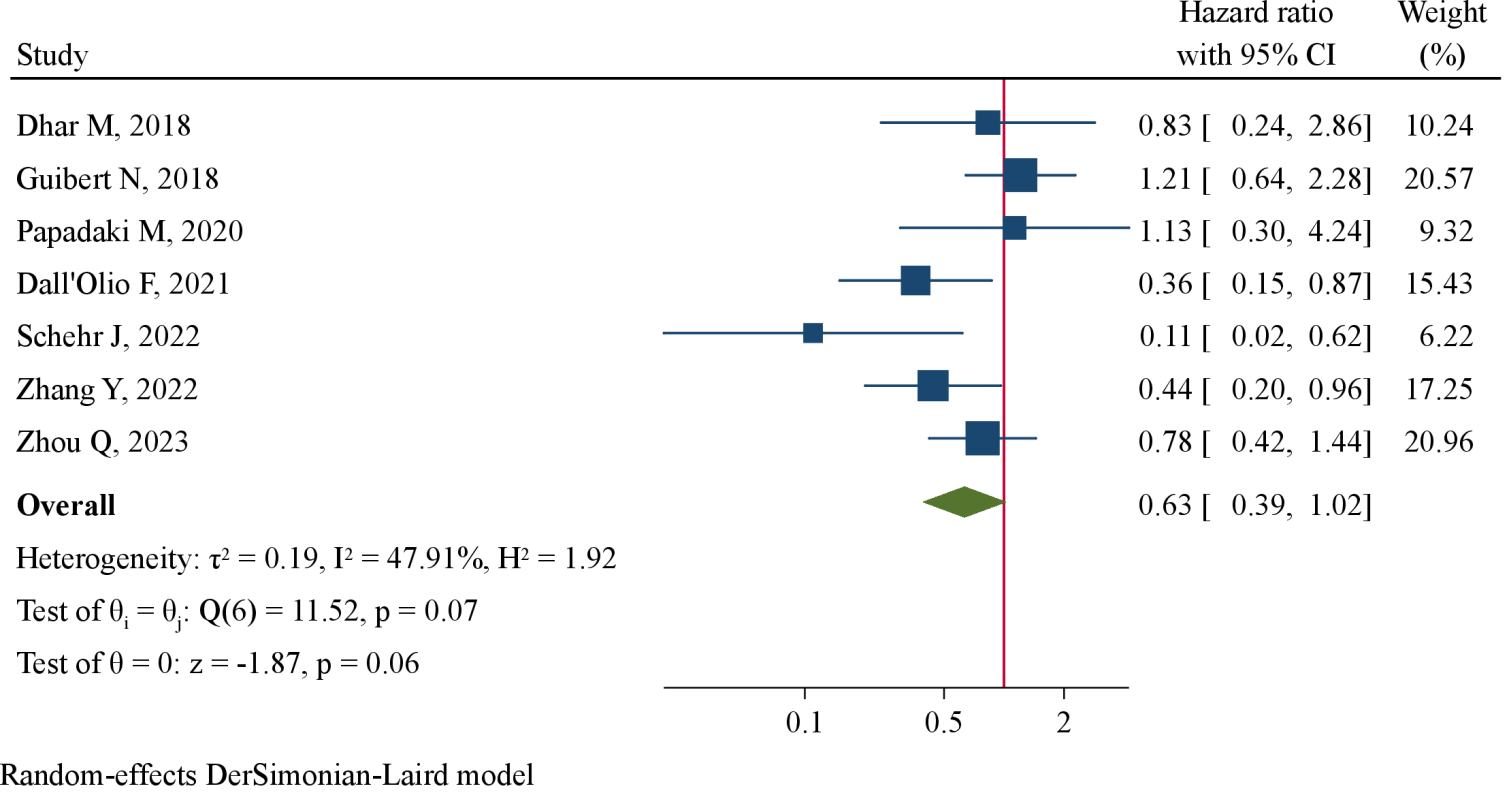
Forest plot of pre-treatment PD-L1^+^ CTCs in association with progression-free survival.

**Figure 5:**
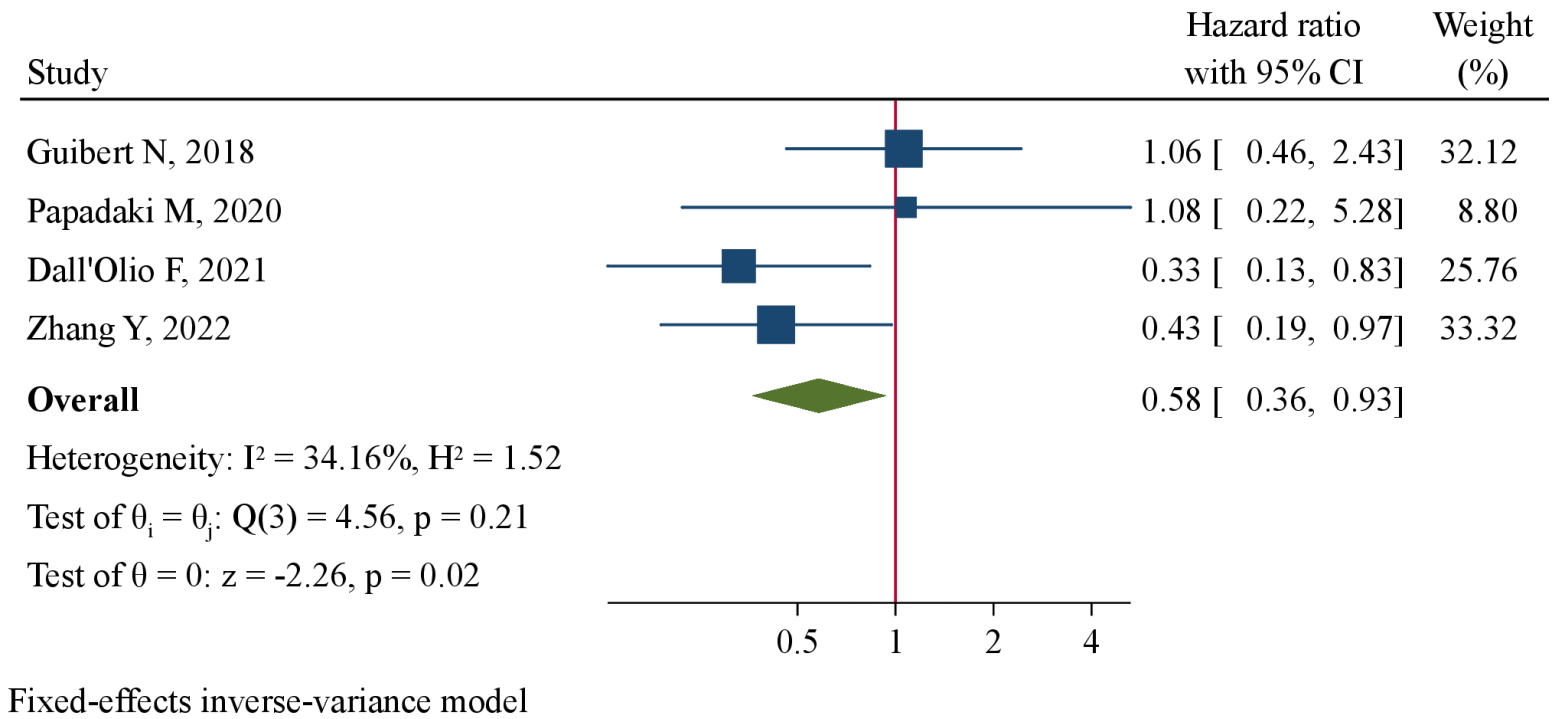
Forest plot of pre-treatment PD-L1^+^ CTCs in association with overall survival.

Meta-analysis encompassing 7 studies (238 patients) using a random-effect model revealed a tendency of pre-treatment PD-L1^+^ CTCs to be correlated with favorable PFS at marginal level of significance (HR=0.63, 95%CI 0.39–1.02, P=0.062, **Figure 4**). By pooling together 4 studies (158 patients), we found significantly improved OS favoring patients with pre-treatment PD-L1^+^ CTCs (HR=0.58, 95%CI 0.36–0.93, P=0.024, **Figure 5**). Subgroup analysis revealed that CTC enrichment method may be the main source of heterogeneity (**Supplementary Table 3**). Pre-treatment PD-L1^+^ CTCs were associated with both improved PFS and OS in studies applying EpCAM-based CTC enrichment (**Supplementary Figure 4**, **5**), whereas not significant association was found in studies implementing size-based CTC enrichment. The between-subgroup differences were statistically significant (P=0.003 and 0.036, respectively).

The prognostic value of post-treatment PD-L1^+^ CTCs and dynamic change of PD-L1^+^ CTCs was both evaluated in only 1 study. Ikeda M et al. found patients with ≥7.7% PD-L1 positivity rate in CTCs had a longer PFS than those with <7.7% positivity rate but did not have OS benefit (30). Spiliotaki M et al. observed longer PFS in patients with decreased PD-L1low CTCs after pembrolizumab treatment (33).

### Prognostic significance of exoPD-L1

**Figure 6:**
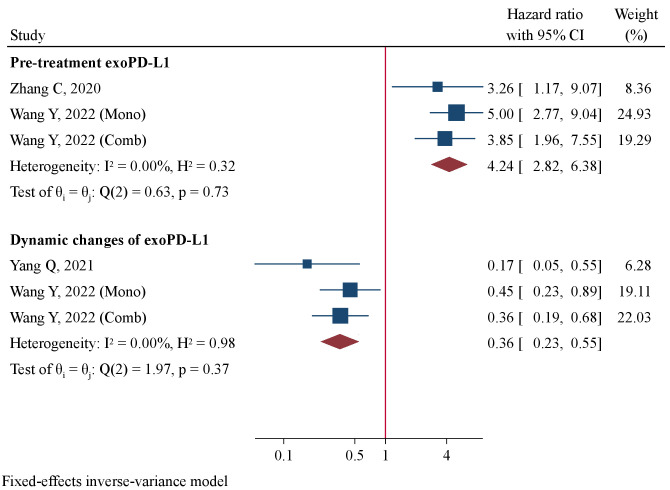
Forest plot of pre-treatment exoPD-L1 levels and dynamic changes in association with progression-free survival.

Two studies comprising 173 patients detected pre-treatment exoPD-L1 levels (16, 34). Wang Y et al. reported results of prognostic value of exoPD-L1 in patients receiving mono-immunotherapy and those with combination immunotherapy separately [34]. They were included in quantitative analysis as two datasets. Meta-analysis using a fixed-effect model revealed patients with high pre-treatment exoPD-L1 concentrations had significantly worse PFS compared to those with low concentrations (HR=4.24, 95%CI 2.82–6.38, P<0.001, **Figure 6**). Two studies involving 170 patients detected dynamic changes of exoPD-L1 levels immunotherapy using near 2-fold up-regulation as cut-offs (34, 35). Patients with up-regulation of exoPD-L1 levels after immunotherapy had significantly favorable PFS than those without obvious up-regulation (HR=0.36, 95%CI 0.23–0.55, P<0.001, **Figure 6**). The correlation between dynamic exoPD-L1 concentrations and OS was reported by only 1 study (35), in which patients with ≥1.86-fold un-regulation had significantly prolonged OS than those with <1.86-fold change (HR=0.24, 95%CI 0.08–0.68, P=0.008).

### Meta-regression analysis of pre-treatment sPD-L1

We conducted meta-regression to analyze whether baseline features modulated the association between pre-treatment sPD-L1 and survival outcomes in NSCLC patients treated with ICIs (**Supplementary Table 4**). Meta-regression showed that median age, percentage of males, percentage of smokers, sample size, percentage of first-line ICIs, and cut-off value did not modulate the association of sPD-L1 with PFS. Moreover, these factors did not modulate the correlation between sPD-L1 and OS except for cut-off value. After excluding an outlier (3357 ng/ml) that was extremely larger than the other cut-offs (27), meta-regression demonstrated a positive linear relationship between cut-off value and effect size (P=0.019, **Supplementary Figure 6**). Cut-off value may partially explain the source of heterogeneity in analysis of pre-treatment sPD-L1 associated with OS.

### Publication bias

Publication bias was assessed by viewing the symmetry of funnel plots and Egger’s test (**Supplementary Table 5**). There was potential publication bias in meta-analysis of dynamic change of sPD-L1 concentrations associated with PFS and OS (P<0.05). Trim-and-fill analysis after imputing 1 and 2 studies showed that the association between dynamic change of sPD-L1 and survivals remained insignificant (PFS: HR=0.86, 95%CI 0.54–1.37; OS: HR=1.00, 95%CI 0.61–1.63; **Supplementary Figure 7**). Thus, publication bias had no obvious impact on results of pooled analysis. We did not observe obvious publication bias in other meta-analyses (**Supplementary Figure 8**).

## Discussion

The interaction between PD-L1 and PD-1 results in cytotoxic T-cell exhaustion and suppresses T-cell immune response that is vital for tumor cell recognition and clearance (45, 46). Inhibitors blocking PD-1/PD-L1 signaling may restore cytotoxic immune response, which are the major and most successful ICIs strategy. The present meta-analysis summarized the prognostic values of several types of PD-L1 blood markers in ICI-treated NSCLC patients. Our study demonstrated that these markers, including pre-treatment sPD-L1, CTC PD-L1 and exoPD-L1, post-treatment sPD-L1, and dynamic changes of exoPD-L1, were significantly associated with survivals. These results suggested PD-L1 blood markers as promising alternative markers to tissue PD-L1 to guide ICIs treatment in NSCLC patients.

sPD-L1 is the cleavage product of membrane-bound form PD-L1 and partially retains PD-1 binding activity. Yet, there seems no significant relationship between sPD-L1 levels and PD-L1 expression on tumor tissue (19, 26, 47). Apart from the proteolytic product, sPD-L1 may also be generated from other sources, including high expression levels of various sPD-L1 isoforms resulted from sPD-L1 alternative splice variants, secretion from dendritic cells, and expression in serum-derived exomes (48–50). Contrary to tissue PD-L1 that predicts better ICIs treatment response and survivals, high pre-treatment sPD-L1 level is associated with worse PFS and OS. One possible explanation is that the abundant sPD-L1 isoforms may neutralize anti-PD-(L)1 blockades in a dosage-dependent manner and inhibit anti-tumor immune function more efficiently (51, 52). Most of the included studies revealed worse survivals in high sPD-L1 group, whereas Zhang S et al. found patients with high sPD-L1 levels tended to have a longer PFS (43). In this study, all patients had been resistant to growth factor receptor tyrosine kinase inhibitors (EGFR-TKIs) and received the anti-PD-1 toripalimab combined with anlotinib (43). It is noted that the association between pre-treatment sPD-L1 and prognosis seems to be tumor-type dependent. The significant associations were only found in NSCLC patients but absent from melanoma and renal cell carcinoma (53, 54).

The relationships of post-treatment sPD-L1 levels and dynamic changes with survivals have also been explored. Himuro H et al. found high levels of sPD-L1 measured 6 weeks after ICIs treatment initiation correlated with worse PFS and OS (41). Zizzari I et al. reported patients with high sPD-L1 levels after 6 cycles of nivolumab treatment had significantly shorter OS (38). Pooled analyses in our study have revealed the clinical significance of post-treatment sPD-L1 in predicting prognosis of NSCLC patients treated with ICIs. Several studies monitored the dynamic changes of sPD-L1 but conclusions regarding the association between up-regulation of sPD-L1 and survivals were inconsistent (19, 23, 29, 31, 35). Oh S et al. found NSCLC patients with >100% increase in sPD-L1 after ICIs treatment had longer PFS and OS, but the results were opposite in melanoma (31). Yet, they could not draw a definite conclusion as most of these studies have very small sample size. Our meta-analysis demonstrated no significant correlation between sPD-L1 dynamic change and survivals in ICI-treated NSCLC patients. A recent patient-level meta-analysis yielded similar conclusion that sPD-L1 changes during ICIs treatment did not influence the prognosis of NSCLC patients regardless of sex, age and ICI type (55).

PD-L1 can be detected on CTCs in many malignant tumors and is correlated with treatment response and prognosis (15, 56–58). Yet, the clinical relevance of CTCs PD-L1 seems to be treatment dependent. PD-L1 expression on CTCs predicted significantly poor survivals in patients treated with non-ICIs treatment but not in ICI-treated patients (59, 60). The present meta-analysis, incorporating more eligible studies, revealed patients with PD-L1^+^ CTCs prior to ICIs treatment had a significantly prolonged OS (HR=0.58, 95%CI 0.36–0.93, P=0.024) and tended to have a longer PFS (HR=0.63, 95%CI 0.39–1.02, P=0.062). Post-treatment PD-L1^+^ CTCs showed clinical significance in patients receiving chemotherapy or radiotherapy, which predicted significantly longer PFS and OS (58, 61). Only one study was performed in NSCLC patients receiving ICIs, showing improved PFS patients with post-treatment PD-L1^+^ CTCs (30). The prognostic value of post-treatment PD-L1+ CTCs and the dynamic change needs investigation in more studies.

Exosomes belong to a subtype of extracellular vesicles with a diameter of 30~150 nm, which can be purified from blood of cancer patients (62). Tumor cells can secret PD-L1 via exosomes and exoPD-L1 exerts immunosuppressive function through inhibiting activation and promoting apoptosis of T cells, suppressing immune memory, and promoting tumor growth (11, 63). Up-regulated exoPD-L1 induces immune escape to promote tumor progression and mediates immunotherapy resistance by competitively binding to anti PD-(L)1 antibody (64). In NSCLC patients treated with immunotherapy, significantly improved PFS was achieved in patients with low pre-treatment exoPD-L1 levels than those with high levels (16, 34). Moreover, the increase of exoPD-L1 levels after ICIs treatment was found to be associated with favorable PFS (34, 35). This increase after several cycles of treatment may reflect successful anti-tumor immunity of immunotherapy as more tissue PD-1/PD-L1 interaction are blocked and more PD-L1 are secreted to exome. Our meta-analysis demonstrated high pre-treatment exoPD-L1 levels predicted poor survival while the increase after treatment predicted better PFS, suggesting exoPD-L1 as biomarker for ICIs treatment.

Our meta-analysis has several strengths than previous ones (53, 65, 66). We focus on NSCLC patients treated with ICIs and include more eligible studies with the largest sample size. Hence, the populations are more homogeneous and the statistical power is larger. Secondly, we evaluate several PD-L1 blood markers at different assessment time-points, i.e. pre-treatment, post-treatment and the dynamic changes. Our study firstly shows significant associations of high post-treatment sPD-L1 levels and pre-treatment PD-L1^+^ CTCs with survivals. Despite promising prognostic value, there are several practical challenges and considerations associated with the clinical implementation of PD-L1 blood markers in ICI-treated NSCLC patient management, and the limitations of our study should be noted. Given the diversity in study features, patients characteristics, ICI types and methodologies of PD-L1 detection, it is inappropriate and difficult to directly translate our findings into clinical practice. The largest obstacles are the PD-L1 assay techniques and cut-off values, which greatly vary among included trials. Standardization efforts, such as the standard protocols for PD-L1 detection and quantification and the optimal and unified PD-L1 thresholds defining who will benefit from ICIs treatment, are urgently warranted to improve the reliability of these markers. Subgroup analyses in our study have provided some clues. We find that high pre-treatment sPD-L1 and exoPD-L1 levels using the optimal cut-off points predict worse survivals than low levels. On the contrary, no significant association is observed between survivals and pre-treatment sPD-L1 levels using median values as cut-off. Therefore, our study suggests that thresholds for sPD-L1 and exoPD-L1 can be established using optimal cut-off points predicting treatment response rather than the median values. For CTC PD-L1, CTC enrichment method seems to be a key factor influencing the prognostic value, of which EpCAM-based enrichment, instead of size-based method, can be recommended. However, these results are obtained from studies with a relative small sample size. Future large-scale, prospective studies are needed to establish the optimal thresholds of PD-L1 and validate the prognostic value in independent cohorts and clinical practice.

## Conclusion

In summary, we observed significantly worse survivals in ICI-treated NSCLC patients with high pre- or post-treaement sPD-L1 levels and pre-treatment exoPD-L1 levels. Moreover, patients with pre-treatment PD-L1^+^ CTCs and those with increasing exoPD-L1 levels after treatment had improved survivals. Therefore, these three PD-L1 blood indicators may be utilized as minimally invasive, convenient, alternative biomarkers to conventional tissue PD-L1 for decision-making and management of ICIs treatment in NSCLC patients.

## Data availability statement

The original contributions presented in the study are included in the article/**Supplementary Material**. Further inquiries can be directed to the corresponding author.

## Author contributions

NZ: Conceptualization, Data curation, Formal Analysis, Writing – original draft, Writing – review & editing. JC: Data curation, Formal Analysis, Writing – original draft, Writing – review & editing. PL: Data curation, Formal Analysis, Writing – original draft, Writing – review & editing. XT: Data curation, Formal Analysis, Writing – original draft, Writing – review & editing. JY: Conceptualization, Supervision, Writing – original draft, Writing – review & editing.
