# Supplementary material for: Prognostic significance of programmed cell death ligand 1 blood markers in non-small cell lung cancer treated with immune checkpoint inhibitors: a systematic review and meta-analysis
Source: Front Immunol. 2024 Jun 10;15:1400262. doi: 10.3389/fimmu.2024.1400262 (PMC11194356; doi:10.3389/fimmu.2024.1400262)
Supplement: Supplementary file 1 [file DataSheet_1.pdf]

Supplementary materials

Supplementary Figures

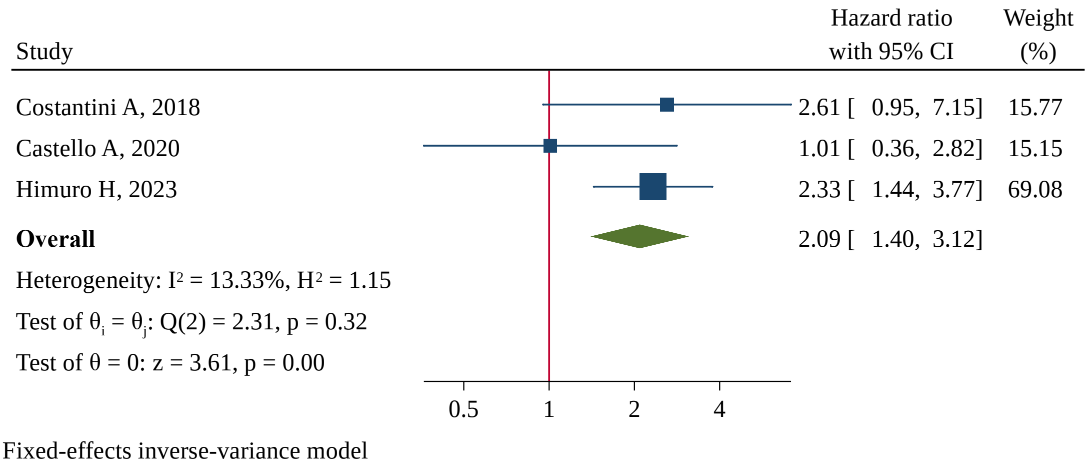

Supplementary Figure 1. Association between post-treatment sPD-L1 levels and PFS. High sPD-L1 levels predicted a shorter PFS.

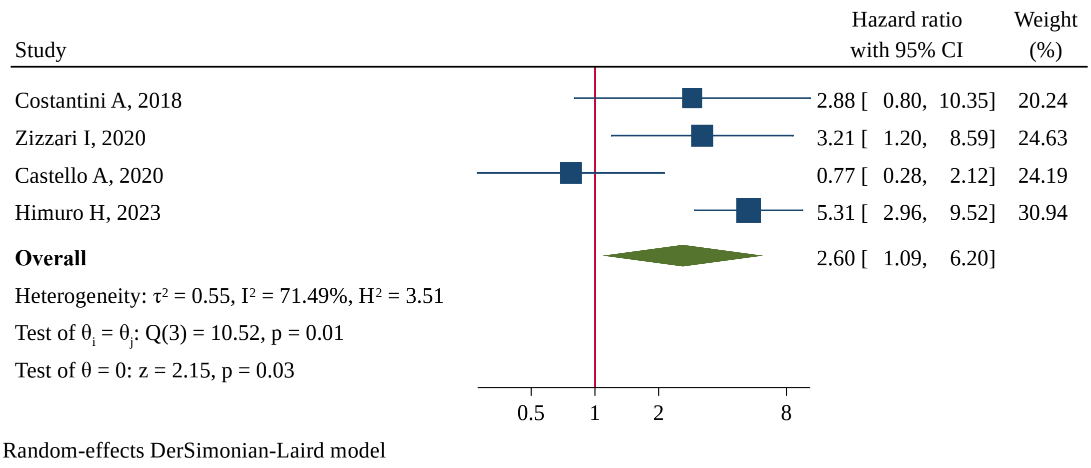

Supplementary Figure 2. Association between post-treatment sPD-L1 levels and OS. High sPD-L1 levels predicted a shorter OS.

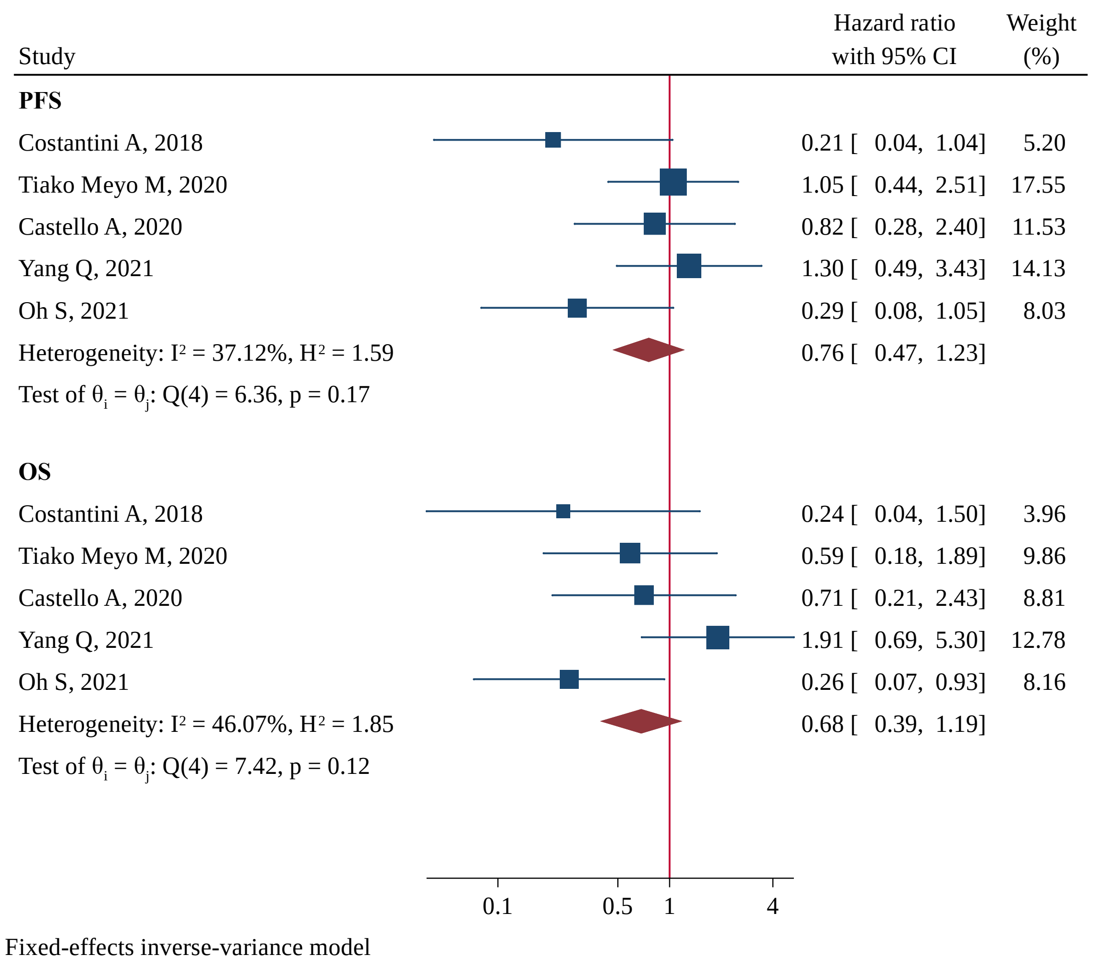

Supplementary Figure 3. Association between dynamic changes of sPD-L1 levels and survivals. Up-regulation of sPD-L1 levels after treatment was not significantly associated with survivals.

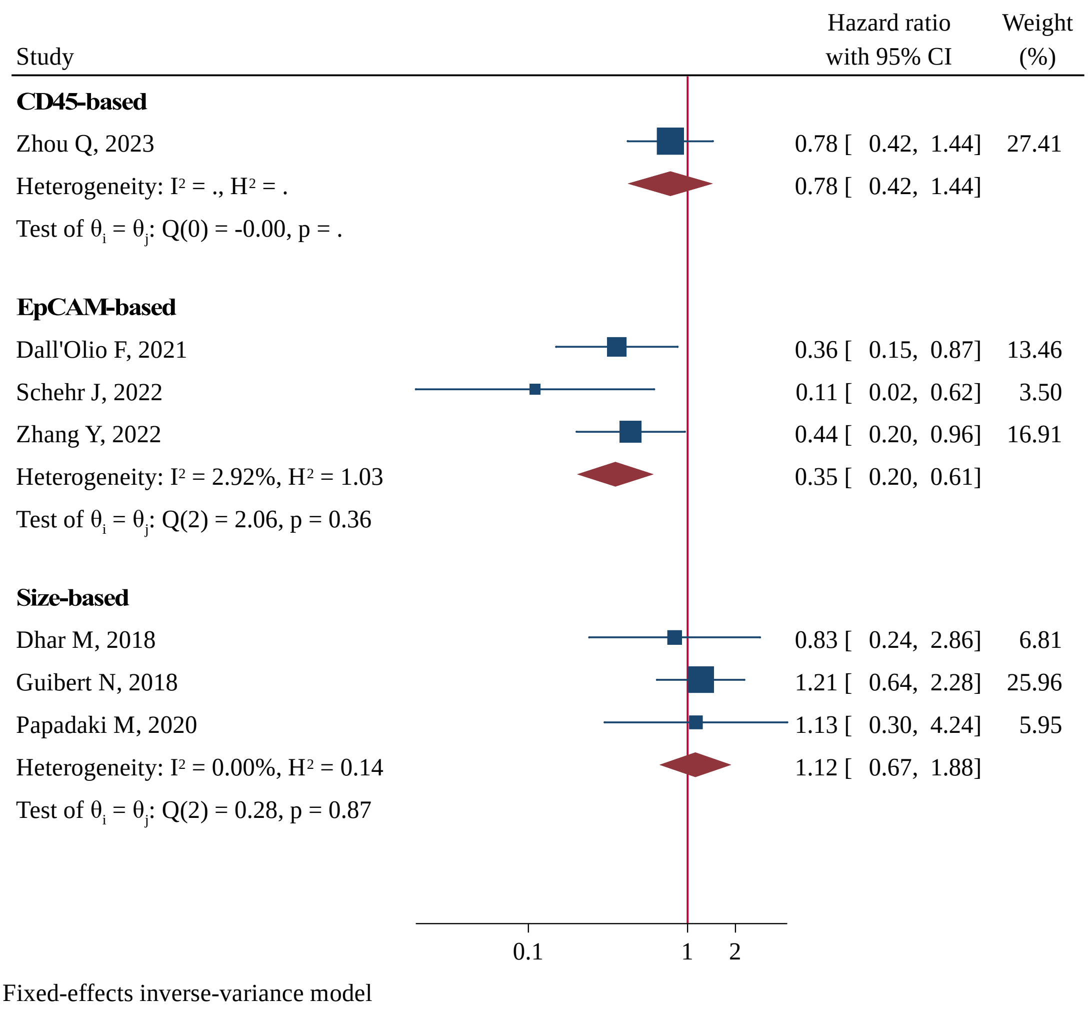

Supplementary Figure 4. Association between pre-treatment CTC PD-L1 and PFS stratified by CTC enrichment method. Pre-treatment PD-L1^{+} CTCs were associated with favorable PFS in EpCAM-based subgroup.

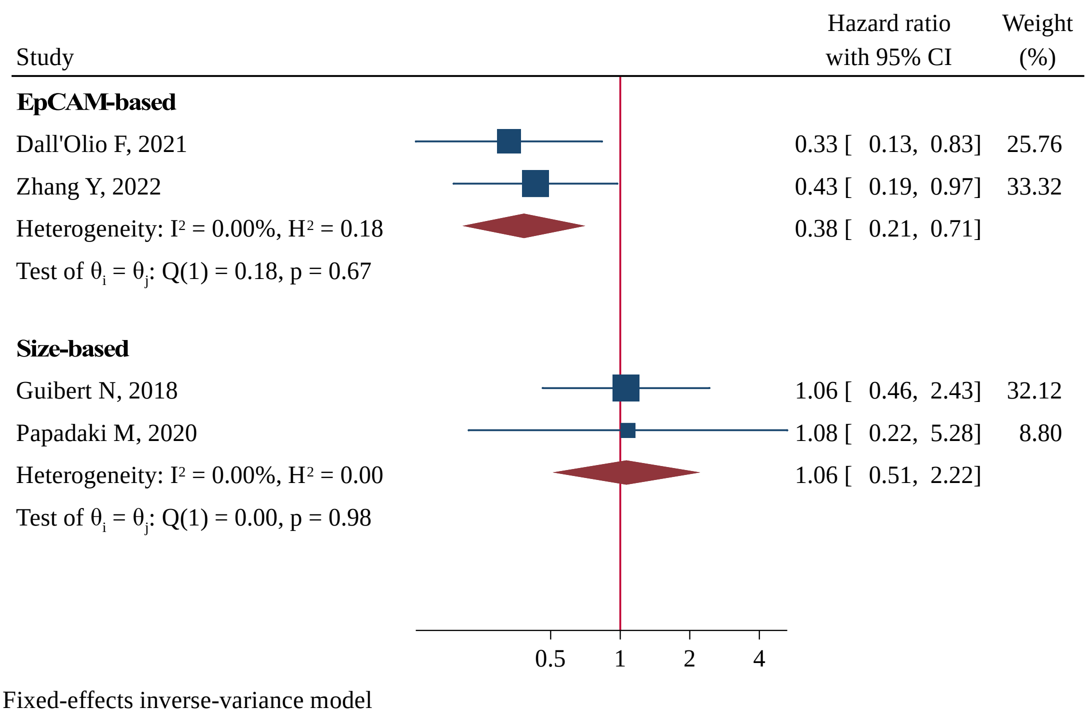

Supplementary Figure 5. Association between pre-treatment CTC PD-L1 and OS stratified by CTC enrichment method. Pre-treatment PD-$L1^+$ CTCs were associated with favorable OS in EpCAM-based subgroup.

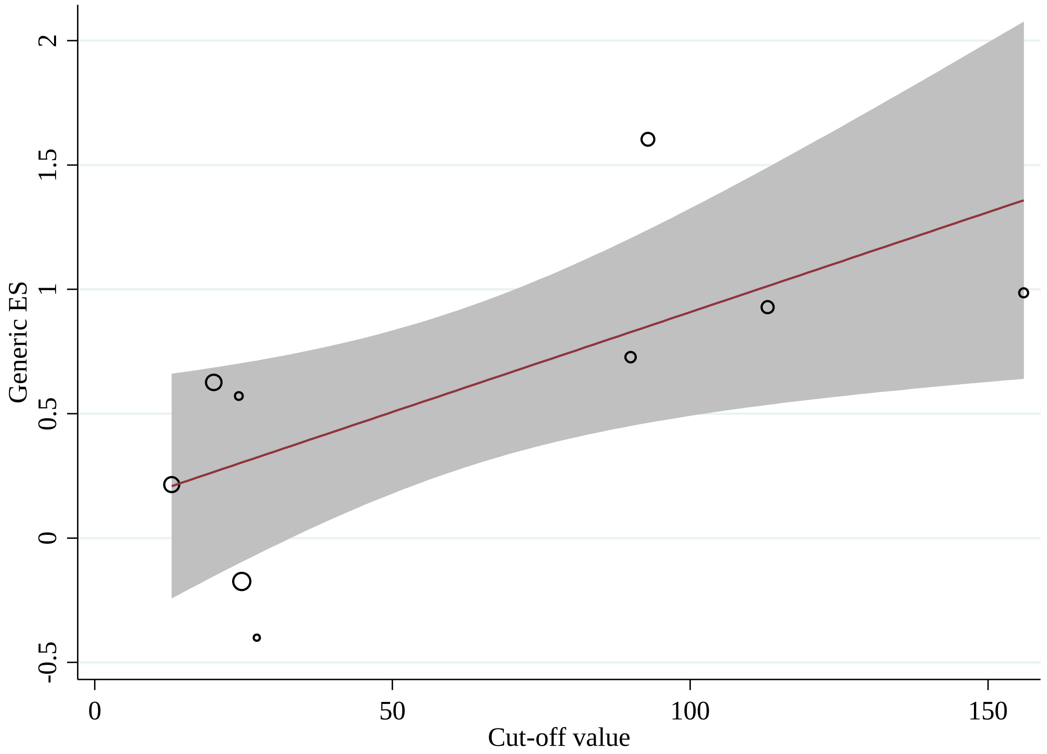

Supplementary Figure 6. Meta-regression analysis of cut-off values in modulating the association between pre-treatment sPD-L1 levels and OS.

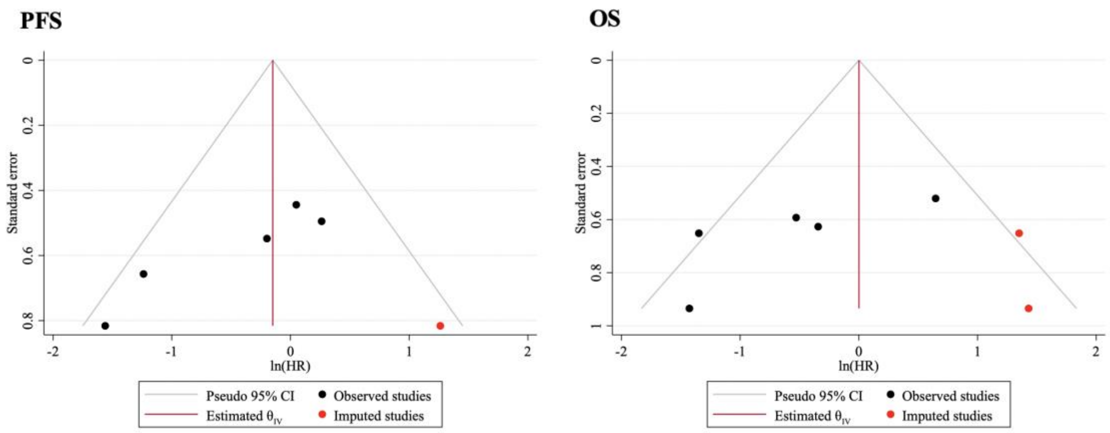

Supplementary Figure 7. Trim-and-fill analysis of dynamic changes of sPD-L1 in association with PFS and OS.

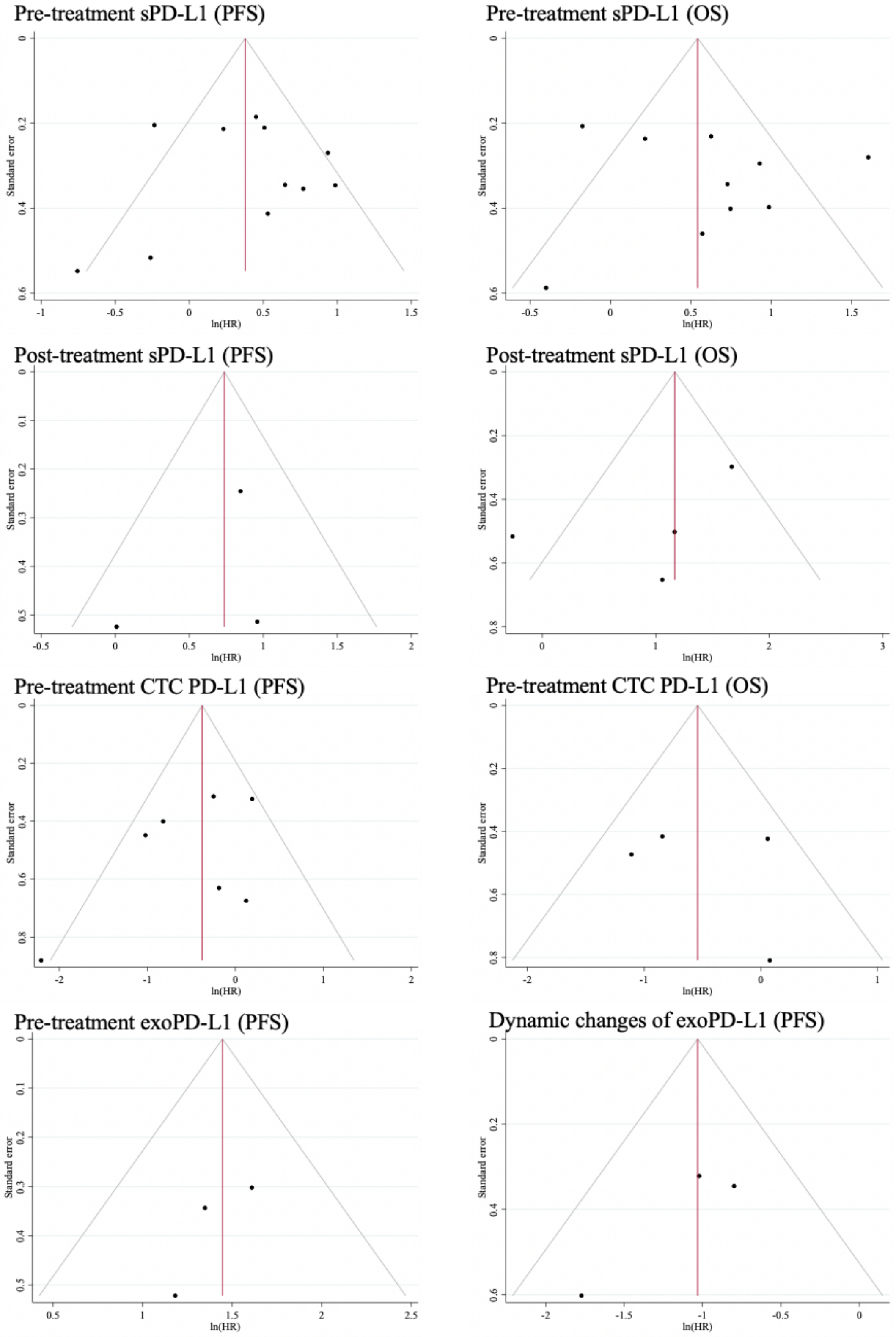

Supplementary Figure 8. Funnel plots of association between PD-L1 blood markers and survival outcomes.
