# Supplementary material for: Prognostic significance of programmed cell death ligand 1 blood markers in non-small cell lung cancer treated with immune checkpoint inhibitors: a systematic review and meta-analysis
Source: Front Immunol. 2024 Jun 10;15:1400262. doi: 10.3389/fimmu.2024.1400262 (PMC11194356; doi:10.3389/fimmu.2024.1400262)
Supplement: Supplementary file 2 [file Table_1.docx]

***Supplementary materials***

**1 PRISMA 2020 Checklist**

| **Section and Topic** | **Item #** | **Checklist item** | **Location where item is reported** |
| --- | --- | --- | --- |
| **TITLE** | | |  |
| Title | 1 | Identify the report as a systematic review. | Title |
| **ABSTRACT** | | |  |
| Abstract | 2 | See the PRISMA 2020 for Abstracts checklist. | Abstract section |
| **INTRODUCTION** | | |  |
| Rationale | 3 | Describe the rationale for the review in the context of existing knowledge. | Paragraph 3 in Introduction |
| Objectives | 4 | Provide an explicit statement of the objective(s) or question(s) the review addresses. | Paragraph 3 in Introduction |
| **METHODS** | | |  |
| Eligibility criteria | 5 | Specify the inclusion and exclusion criteria for the review and how studies were grouped for the syntheses. | “Inclusion and exclusion criteria" section |
| Information sources | 6 | Specify all databases, registers, websites, organisations, reference lists and other sources searched or consulted to identify studies. Specify the date when each source was last searched or consulted. | “Literature search” section |
| Search strategy | 7 | Present the full search strategies for all databases, registers and websites, including any filters and limits used. | “Literature search” section |
| Selection process | 8 | Specify the methods used to decide whether a study met the inclusion criteria of the review, including how many reviewers screened each record and each report retrieved, whether they worked independently, and if applicable, details of automation tools used in the process. | “Data extraction” section |
| Data collection process | 9 | Specify the methods used to collect data from reports, including how many reviewers collected data from each report, whether they worked independently, any processes for obtaining or confirming data from study investigators, and if applicable, details of automation tools used in the process. | “Data extraction” section |
| Data items | 10a | List and define all outcomes for which data were sought. Specify whether all results that were compatible with each outcome domain in each study were sought (e.g. for all measures, time points, analyses), and if not, the methods used to decide which results to collect. | “Data extraction” section |
|  | 10b | List and define all other variables for which data were sought (e.g. participant and intervention characteristics, funding sources). Describe any assumptions made about any missing or unclear information. | “Data extraction” section |
| Study risk of bias assessment | 11 | Specify the methods used to assess risk of bias in the included studies, including details of the tool(s) used, how many reviewers assessed each study and whether they worked independently, and if applicable, details of automation tools used in the process. | “Quality assessment of included studies” section |
| Effect measures | 12 | Specify for each outcome the effect measure(s) (e.g. risk ratio, mean difference) used in the synthesis or presentation of results. | “Statistical analysis” section |
| Synthesis methods | 13a | Describe the processes used to decide which studies were eligible for each synthesis (e.g. tabulating the study intervention characteristics and comparing against the planned groups for each synthesis (item #5)). | “Statistical analysis” section |
|  | 13b | Describe any methods required to prepare the data for presentation or synthesis, such as handling of missing summary statistics, or data conversions. | “Statistical analysis” section |
|  | 13c | Describe any methods used to tabulate or visually display results of individual studies and syntheses. | “Statistical analysis” section |
|  | 13d | Describe any methods used to synthesize results and provide a rationale for the choice(s). If meta-analysis was performed, describe the model(s), method(s) to identify the presence and extent of statistical heterogeneity, and software package(s) used. | “Statistical analysis” section |
|  | 13e | Describe any methods used to explore possible causes of heterogeneity among study results (e.g. subgroup analysis, meta-regression). | “Statistical analysis” section |
|  | 13f | Describe any sensitivity analyses conducted to assess robustness of the synthesized results. | “Statistical analysis” section |
| Reporting bias assessment | 14 | Describe any methods used to assess risk of bias due to missing results in a synthesis (arising from reporting biases). | NA |
| Certainty assessment | 15 | Describe any methods used to assess certainty (or confidence) in the body of evidence for an outcome. | NA |
| **RESULTS** | | |  |
| Study selection | 16a | Describe the results of the search and selection process, from the number of records identified in the search to the number of studies included in the review, ideally using a flow diagram. | “Baseline characteristics” section |
|  | 16b | Cite studies that might appear to meet the inclusion criteria, but which were excluded, and explain why they were excluded. | Supplementary Table 1 |
| Study characteristics | 17 | Cite each included study and present its characteristics. | P6-7, Table 1, Supplementary Table 2 |
| Risk of bias in studies | 18 | Present assessments of risk of bias for each included study. | P6, Table 1 |
| Results of individual studies | 19 | For all outcomes, present, for each study: (a) summary statistics for each group (where appropriate) and (b) an effect estimate and its precision (e.g. confidence/credible interval), ideally using structured tables or plots. | P7-9, Figure 2-6, Supplementary Figure 1-3 |
| Results of syntheses | 20a | For each synthesis, briefly summarise the characteristics and risk of bias among contributing studies. | NA |
|  | 20b | Present results of all statistical syntheses conducted. If meta-analysis was done, present for each the summary estimate and its precision (e.g. confidence/credible interval) and measures of statistical heterogeneity. If comparing groups, describe the direction of the effect. | Prognostic significance of sPD-L1, CTC PD-L1, exoPD-L1 sections. |
|  | 20c | Present results of all investigations of possible causes of heterogeneity among study results. | “Meta-regression analysis” section, Table 2, Supplementary table 3 |
|  | 20d | Present results of all sensitivity analyses conducted to assess the robustness of the synthesized results. | NA |
| Reporting biases | 21 | Present assessments of risk of bias due to missing results (arising from reporting biases) for each synthesis assessed. | NA |
| Certainty of evidence | 22 | Present assessments of certainty (or confidence) in the body of evidence for each outcome assessed. | NA |
| **DISCUSSION** | | |  |
| Discussion | 23a | Provide a general interpretation of the results in the context of other evidence. | Paragraph 1 in Discussion |
|  | 23b | Discuss any limitations of the evidence included in the review. | Paragraph 6 in Discussion |
|  | 23c | Discuss any limitations of the review processes used. | Paragraph 6 in Discussion |
|  | 23d | Discuss implications of the results for practice, policy, and future research. | “Conclusion” section |
| **OTHER INFORMATION** | | |  |
| Registration and protocol | 24a | Provide registration information for the review, including register name and registration number, or state that the review was not registered. | NA |
|  | 24b | Indicate where the review protocol can be accessed, or state that a protocol was not prepared. | NA |
|  | 24c | Describe and explain any amendments to information provided at registration or in the protocol. | NA |
| Support | 25 | Describe sources of financial or non-financial support for the review, and the role of the funders or sponsors in the review. | Funding |
| Competing interests | 26 | Declare any competing interests of review authors. | Conflict of interest |
| Availability of data, code and other materials | 27 | Report which of the following are publicly available and where they can be found: template data collection forms; data extracted from included studies; data used for all analyses; analytic code; any other materials used in the review. | Data availability statement |

**2 Supplementary Tables**

Supplementary Table 1. List of excluded studies after reviewing full-text.

| Study | Title | Marker | Reason for exclusion | PMID |
| --- | --- | --- | --- | --- |
| Nicolazzo C, 2016 | Monitoring PD-L1 positive circulating tumor cells in non-small cell lung cancer patients treated with the PD-1 inhibitor Nivolumab | CTC PD-L1 | Not investigating survival outcomes | 27553175 |
| Kulashinghe A, 2018 | The prognostic significance of circulating tumor cells in head and neck and non-small-cell lung cancer | CTC PD-L1 | Patients received various treatments including ICIs | 30565869 |
| Janning M, 2019 | Determination of PD-L1 Expression in Circulating Tumor Cells of NSCLC Patients and Correlation with Response to PD-1/PD-L1 Inhibitors | CTC PD-L1 | Patients received various treatments including ICIs | 31212989 |
| Ando K, 2019 | Plasma Levels of Soluble PD-L1 Correlate With Tumor Regression in Patients With Lung and Gastric Cancer Treated With Immune Checkpoint Inhibitors | sPD-L1 | Investigating the association of sPD-L1 with survivals after ICI treatment in NSCLC and gastric cancer. The results for NSCLC cohort are not provided | 31519633 |
| Zhang L, 2020 | PD-L1+ aneuploid circulating tumor endothelial cells (CTECs) exhibit resistance to the checkpoint blockade immunotherapy in advanced NSCLC patients | CTC PD-L1 | Performing survival analysis at specimen level | 31678168 |
| He Y, 2020 | Study on the Expression Levels and Clinical Significance of PD-1 and PD-L1 in Plasma of NSCLC Patients | sPD-L1 | Patients received non-ICI treatment | 32168233 |
| Cheng Y, 2020 | Detection of PD-L1 Expression and Its Clinical Significance in Circulating Tumor Cells from Patients with Non-Small-Cell Lung Cancer | CTC PD-L1 | Treatment is unknown | 32256114 |
| Del Re M, 2021 | A multiparametric approach to improve the prediction of response to immunotherapy in patients with metastatic NSCLC | exoPD-L1 | Detecting PD-L1 mRNA expression in exosomes | 33315149 |
| Shimada Y, 2021 | Serum-derived exosomal PD-L1 expression to predict anti-PD-1 response and in patients with non-small cell lung cancer | exoPD-L1 | Not investigating survival outcomes | 33837261 |
| Wang H, 2021 | Heterogeneity and prognosis of programmed cell death-ligand 1 expression in the circulating tumor cells of non-small cell lung cancer | CTC PD-L1 | Patients received non-ICI treatment | 34097427 |
| Sinoquet L, 2021 | Programmed Cell Death Ligand 1-Expressing Circulating Tumor Cells: A New Prognostic Biomarker in Non-Small Cell Lung Cancer | CTC PD-L1 | Patients received non-ICI treatment | 34355741 |
| Tan Z, 2021 | Assessment of PD-L1 Expression on Circulating Tumor Cells for Predicting Clinical Outcomes in Patients with Cancer Receiving PD-1/PD-L1 Blockade Therapies | CTC PD-L1 | Investigating the association of CTC PD-L1 with survivals after ICI treatment in various cancers not including lung cancer | 34516729 |
| Katsarou S, 2022 | Detyrosinated α-Tubulin, Vimentin and PD-L1 in Circulating Tumor Cells (CTCs) Isolated from Non-Small Cell Lung Cancer (NSCLC) Patients | CTC PD-L1 | Patients received non-ICI treatment | 35207643 |
| Kennedy L, 2022 | Liquid Biopsy Assessment of Circulating Tumor Cell PD-L1 and IRF-1 Expression in Patients with Advanced Solid Tumors Receiving Immune Checkpoint Inhibitor | CTC PD-L1 | Investigating the association of CTC PD-L1 with survivals after ICI treatment in various cancers including NSCLC. The results for NSCLC cohort are not available | 35696014 |
| Inoue Y, 2022 | Serum immune modulators associated with immune-related toxicities and efficacy of atezolizumab in patients with non-small cell lung cancer | sPD-L1 | The results for sPD-L1 in association with survival are not provided | 35834011 |
| Zhang Z, 2022 | Blood exosome PD-L1 is associated with PD-L1 expression measured by immunohistochemistry, and lymph node metastasis in lung cancer | exoPD-L1 | Not investigating survival outcomes | 36228365 |
| Akbar S, 2023 | Circulating exosomal immuno-oncological checkpoints and cytokines are potential biomarkers to monitor tumor response to anti-PD-1/PD-L1 therapy in non-small cell lung cancer patients | exoPD-L1 | Not investigating survival outcomes | 36741391 |
| Cohen E, 2023 | Gene expression profiling of circulating tumor cells captured by MicroCavity Array is superior to enumeration in demonstrating therapy response in patients with newly diagnosed advanced and locally advanced non-small cell lung cancer | CTC PD-L1 | Patients received non-ICI treatment | 36762061 |
| Cheng Y, 2023 | A Study on the Clinical Significance of Blood Exosomal PD-L1 in Non-Small Cell Lung Cancer Patients and its Correlation with PD-L1 in Tumor Tissues | exoPD-L1 | Not investigating survival outcomes | 37459865 |
| Zhu H, 2023 | Analysis of soluble programmed death-1 ligand-1 of lung cancer patients with different characteristics | sPD-L1 | Patients received non-ICI treatment | 37782182 |
| Eslami-S Z, 2023 | Circulating tumour cells and PD-L1-positive small extracellular vesicles: the liquid biopsy combination for prognostic information in patients with metastatic non-small cell lung cancer | exoPD-L1 | Whether patients received ICI treatment is unknown | 37973956 |

Supplementary Table 2. Other information regarding PD-L1 detection, cut-off determination, HR extraction, and follow-up of studies included in meta-analysis.

| Study | CTC enrichment | PD-L1 detection and kits | Cut-off value | Cut-off determination | HR analysis | HR extraction | Median follow-up, month |
| --- | --- | --- | --- | --- | --- | --- | --- |
| **sPD-L1 (pre- or post-treatment)** | | | | | | | |
| Okuma Y, 2018 | - | ELISA (Cloud-Clone) | 3.357 ng/ml | Optimal cut-off point of ROC curve to predict response to treatment | Univariate | Estimated | NA |
| Costantini A, 2018 | - | ELISA (Abcam) | 33.97 pg/ml | Optimal cut-off point of ROC curve to predict response to treatment | Univariate | Estimated | 16.3 (IQR 11.7-21.1) |
| Tiako Meyo M, 2020 | - | ELISA (Cloud-Clone) | 156 pg/ml | Lower limit of quantification | Univariate | Reported | 26.8 (IQR 18.4-37.1) |
| Castello A, 2020 | - | ELISA (R&D Systems) | 27.22 pg/ml | Median value | Univariate | Estimated | 10.3 (range 2-29) |
| Mazzaschi G, 2020 | - | ELISA (R&D Systems) | 113 pg/ml | Optimal cut-off point by CART analysis | Univariate | Reported | 17.3 |
| Murakami S, 2020 | - | ELISA (R&D Systems) | 90 pg/ml | Mean value plus 2 standard deviation of healthy volunteers | Multivariate | Reported | NA |
| Zizzari I, 2020 | - | Luminex multiplex assay | 20 pg/ml | Median value | Univariate | Estimated | NA |
| Zamora Atenza C, 2022 | - | ELISA (Invitrogen) | 12.94 pg/ml | Optimal cut-off point of ROC curve to discriminate patients with progressive vs no-progressive disease | Univariate | Reported | 10.97 (IQR 6.06-21.87) |
| Genova C, 2023 (PC) | - | ELISA (Abcam) | 24.2 pg/ml | Median value | Multivariate | Reported | 12.7 (range 0.27-43.4) |
| Genova C, 2023 (NC) | - | ELISA (Abcam) | 24.7 pg/ml | Median value | Multivariate | Reported | 8.7 (range 0.53-70.9) |
| Yi L, 2023 | - | ELISA (non-commercial) | 280.64 pg/ml | Median value | Univariate | Estimated | NA |
| Himuro H, 2023 | - | ELISA (R&D Systems) | 55.3 pg/ml for PFS, 92.9 pg/ml for OS | Optimal cut-off point with the most significant split by a log-rank test | Univariate | Estimated | NA |
| Zhang S, 2023 | - | ECL | 137.2681 pg/ml | 70% percentile | Univariate | Estimated | NA |
| Chmielewska I, 2023 | - | ELISA (ThermoFisher) | 20 pg/ml | Median value | Univariate | Reported | NA |
| **sPD-L1 (dynamic change)** | | | | | | | |
| Costantini A, 2018 | - | ELISA (Abcam) | Increase | NA | Multivariate | Reported | 16.3 (IQR 11.7-21.1) |
| Tiako Meyo M, 2020 | - | ELISA (Cloud-Clone) | Increase | NA | Univariate | Reported | 26.8 (IQR 18.4-37.1) |
| Castello A, 2020 | - | ELISA (R&D Systems) | Increase | NA | Univariate | Estimated | 10.3 (range 2-29) |
| Yang Q, 2021 | - | ELISA (R&D Systems) | 0.95-fold change | NA | Univariate | Reported | NA |
| Oh S, 2021 | - | ELISA (Invitrogen) | 1-fold change | NA | Univariate | Estimated | NA |
| **CTCs PD-L1 (pre- or post-treatment)** | | | | | | | |
| Dhar M, 2018 | Size-based (Vortex HT Chip) | IF | 2 PD-L1+ CTCs^#^ | NA | Univariate | Reported | NA |
| Guibert N, 2018 | Size-based (ISET) | IF | 1% PD-L1+ CTCs^&^ | Presence of PD-L1+ CTC | Univariate | Reported | NA |
| Papadaki M, 2020 | Size-based (Parsortix) | IF | 1 PD-L1+ CTC^&^ | Presence of PD-L1+ CTC | Univariate | Estimated | NA |
| Dall'Olio F, 2021 | EpCAM-based (CellSearch) | IF | 1 PD-L1+ CTC^&^ | Presence of PD-L1+ CTC | Univariate | Reported | 12 (95%CI 5.2-18.8) |
| Ikeda M, 2021 | Size-based (MCA) | IF | 7.7% PD-L1+ CTCs^#^ | Optimal cut-off point of ROC curve to segregate DCB from non-DCB | Univariate | Estimated | NA |
| Schehr J, 2022 | EpCAM-based | IF | 1.5 logMFI^#^ | Optimal cut-off point of ROC curve to segregate DCB from non-DCB | Univariate | Reported | NA |
| Zhang Y, 2022 | EpCAM-based (IsoFlux) | IF | 32.5% PD-L1+ CTCs^#^ | Median value | Univariate | Reported | 26.5 |
| Zhou Q, 2023 | CD45-based | IF | 1 PD-L1+ CTC^&^ | Presence of PD-L1+ CTC | Univariate | Estimated | NA |
| **CTCs PD-L1 (dynamic change)** | | | | | | | |
| Spliliotaki 2022 | Size-based | IF | Increase | NA | Univariate | Estimated | NA |
| **exoPD-L1 (pre- or post-treatment)** | | | | | | | |
| Zhang C, 2020 | - | ELISA (R&D Systems) | 149 pg/ml | Optimal cut-off point | Univariate | Estimated | NA |
| Wang Y, 2022 | - | ELISA (Invitrogen) | 0.54 ng/ml (monotherapy), 0.55 ng/ml (combination therapy) | Optimal cut-off point | Univariate | Reported | NA |
| **exoPD-L1 (dynamic change)** | | | | | | | |
| Yang Q, 2021 | - | SIMOA (Quanterix) | 1.86-fold change | Optimal cut-off point of ROC curve to predict response to treatment | Univariate | Reported | NA |
| Wang Y, 2022 | - | ELISA (Invitrogen) | 1.96-fold change (monotherapy), 2.08 (combination therapy) | Optimal cut-off point of ROC curve to predict response to treatment | Univariate | Reported | NA |

^#^ Defining high or low levels of PD-L1+ CTCs.

^&^ Defining the presence or absence of PD-L1+ CTCs.

CART: Classification and regression tree; CTC: circulating tumor cell; DCB: durable clinical benefit; ECL: electrochemiluminescence; ELISA: enzyme-Linked immunosorbent assay; HR: hazard ratio; ICIs: immune checkpoint inhibitors; IF: Immunofluorescence; IQR: interquartile range; MFI: mean fluorescence intensity; NA: not available; ROC: receiver operating characteristic.

Supplementary Table 3. Subgroup analysis of dynamic change of sPD-L1 and pre-treatment CTC PD-L1 in association with survival outcomes.

| Subgroup | PFS | | | | |  | OS | | | | |
| --- | --- | --- | --- | --- | --- | --- | --- | --- | --- | --- | --- |
|  | N | I^2^, % | HR (95%CI) | P1 | P2 |  | N | I^2^, % | HR (95%CI) | P1 | P2 |
| **Dynamic change of sPD-L1** | | |  |  |  |  |  |  |  |  |  |
| Region |  |  |  |  | 0.996 |  |  |  |  |  | 0.778 |
| East Asia | 2 (38) | 70.0 | 0.65 (0.15-2.83) | 0.569 |  |  | 2 (38) | 82.5 | 0.73 (0.10-5.16) | 0.755 |  |
| Europe or America | 3 (101) | 34.1 | 0.76 (0.41-1.41) | 0.381 |  |  | 3 (101) | 0 | 0.54 (0.25-1.17) | 0.117 |  |
| HR extraction |  |  |  |  | 0.310 |  |  |  |  |  | 0.460 |
| Reported | 3 (102) | 48.1 | 0.91 (0.50-1.65) | 0.753 |  |  | 3 (102) | 56.5 | 0.77 (0.25-2.37) | 0.645 |  |
| Estimated | 2 (37) | 32.3 | 0.54 (0.24-1.22) | 0.137 |  |  | 2 (37) | 19.0 | 0.44 (0.18-1.06) | 0.068 |  |
| **Pre-treatment CTC PD-L1** | |  |  |  |  |  |  |  |  |  |  |
| Region |  |  |  |  | 0.996 |  |  |  |  |  | - |
| East Asia | 2 (79) | 21.0 | 0.63 (0.39-1.02) | 0.059 |  |  | 1 (30) | - | - | - |  |
| Europe or America | 5 (159) | 60.1 | 0.62 (0.29-1.32) | 0.215 |  |  | 3 (128) | 46.8 | 0.68 (0.38-1.20) | 0.185 |  |
| CTC enrichment |  |  |  |  | 0.003^#^ |  |  |  |  |  | 0.036 |
| Size-based | 3 (121) | 0 | 1.12 (0.67-1.88) | 0.667 |  |  | 2 (104) | 0 | 1.06 (0.51-2.22) | 0.868 |  |
| EpCAM-based | 3 (68) | 2.9 | 0.35 (0.20-0.61) | <0.001 |  |  | 2 (54) | 0 | 0.38 (0.21-0.71) | 0.002 |  |
| CD45-based | 1 (49) | - | - | - |  |  | 0 | - | - | - |  |
| Cut-off value |  |  |  |  | 0.086 |  |  |  |  |  | - |
| 1 PD-L1+ CTC | 4 (177) | 40.8 | 0.82 (0.56-1.19) | 0.289 |  |  | 3 (128) | 46.8 | 0.68 (0.38-1.20) | 0.185 |  |
| Other values | 3 (61) | 42.7 | 0.43 (0.23-0.80) | 0.008 |  |  | 1 (30) | - | - | - |  |

^#^ P value for comparison between size-based subgroup to EpCAM-based subgroup.

P1: p value for pooled analysis. P2: p value for between-subgroup comparison.

Supplementary Table 4. Meta-regression analysis of baseline variables modulating the association of pre-treatment sPD-L1 with survival outcomes.

| Variables | Coefficient | Standard error | z | P |
| --- | --- | --- | --- | --- |
| PFS |  |  |  |  |
| Median age | 0.008 | 0.041 | 0.19 | 0.847 |
| %Male | 1.06 | 1.39 | 0.76 | 0.448 |
| %Smokers | 1.35 | 0.92 | 1.46 | 0.145 |
| Sample size | 0.0015 | 0.0027 | 0.55 | 0.581 |
| %First line | 0.48 | 0.48 | 1.02 | 0.308 |
| Cut-off value | 0.0018 | 0.0019 | 0.94 | 0.346 |
| OS |  |  |  |  |
| Median age | -0.023 | 0.06 | -0.39 | 0.700 |
| %Male | -0.59 | 2.85 | -0.20 | 0.845 |
| %Smokers | -3.04 | 2.43 | -1.25 | 0.210 |
| Sample size | 0.0013 | 0.0036 | 0.35 | 0.730 |
| %First line | 0.36 | 0.65 | 0.56 | 0.577 |
| Cut-off value^#^ | 0.008 | 0.0034 | 2.35 | 0.019 |

^#^ Excluding an outlier.

Supplementary Table 5. Egger’s test for publication bias.

| Marker | z | P |
| --- | --- | --- |
| Pre-treatment sPD-L1 |  |  |
| PFS | -0.65 | 0.515 |
| OS | -0.21 | 0.837 |
| Post-treatment sPD-L1 |  |  |
| PFS | -0.83 | 0.409 |
| OS | -0.81 | 0.420 |
| Dynamic change of sPD-L1 |  |  |
| PFS | -2.24 | 0.020 |
| OS | -2.07 | 0.038 |
| Pre-treatment PD-L1^+^ CTCs |  |  |
| PFS | -1.27 | 0.204 |
| OS | 0.56 | 0.577 |
| Pre-treatment exoPD-L1 |  |  |
| PFS | -0.68 | 0.500 |
| Dynamic change of exoPD-L1 |  |  |
| PFS | -1.25 | 0.210 |
